# Special Issue “Recommendations for Clinical Management of Glaucoma”

**DOI:** 10.3390/jcm11061499

**Published:** 2022-03-09

**Authors:** Paolo Fogagnolo

**Affiliations:** 1 Department of Health Sciences, Università degli Studi di Milano, Via Antonio di Rudini’, 8, 20142 Milan, Italy; paolo.fogagnolo@unimi.it; 2 Eye Unit, ASST Santi Paolo e Carlo—San Paolo Hospital, University of Milan, 20143 Milan, Italy

Glaucoma is a group of eye conditions that damage the optic nerve head and affect visual function, potentially leading to blindness. These conditions include adult and child chronic forms as well as acute forms. The prevalence of glaucoma is increasing: currently, about 60 million people are affected worldwide, and this number is expected to increase up to 110 million by 2040 [1]. 

The scene of glaucoma diagnosis and management is rapidly changing. A great number of innovations and technological developments have advanced over the last two decades, and this is reflected in the publication trends in the field: 1299 papers were published in 2001, compared with 4665 in 2021 (search performed on 3 March 2022 on https://pubmed.ncbi.nlm.nih.gov using “glaucoma” as the keyword). To address these changes, this Special Issue in the *Journal of Clinical Medicine (JCM)* is dedicated to collecting high-quality scientific contributions focusing on diagnostic and clinical modifications of glaucoma management.

Four studies focused on new methods used to improve glaucoma diagnosis. Considering the increasing prevalence of glaucoma and the limited resources available in many countries, screening procedures may be helpful in reducing the unacceptably high rate of missed detections in the disease. Our group published a paper comparing two screening procedures (quick and standard strategies) using the Compass fundus automated perimeter [2]. The standard grid tested 52 locations, whereas the quick strategy only tested 24 locations. We showed that the two tests achieve similar diagnostic performances and that the quick strategy guarantees 60% time savings compared with the standard grid (mean test duration was 40 seconds in normal subjects and 71 seconds in patients with glaucoma). Moreover, the Compass device automatically collects true-color images of the optic nerve head, which can be used to further improve the diagnostic performance; the instrument can be easily used also in telemedicine settings. Finally, this study has the merit of stimulating discussions on the ideal grid patterns to be used in perimetry. On a previous paper, we showed that a denser macular grid allows for a better follow-up on patients with perimetric glaucoma [3]; here, we showed that screening tests obtain similar performances even though more than half of the locations are not evaluated. The number of locations must be tailored to the different clinical settings in order to obtain better balance between the test time and the detection of glaucoma defects and their progression.

The following three papers focused on the diagnostic performance on specific forms of glaucoma, also analyzing the impact of risk factors. Dr. Stingl and coauthors published the first data from a German Registry of childhood glaucoma [4]. They prospectively evaluated the phenotype–genotype association on 29 patients and identified novel variants of genetic mutations and showed how consanguinity is an important risk factor in these patients. Moreover, this study has the merit of highlighting the importance of obtaining high-quality prospective data using standardized procedures, as high as those shared into registries. For a mean of 5 years, Dr. Lee and coauthors studied a cohort of patients with myopia and chronic glaucoma who had received refractive surgery or not [5]. This topic is extremely relevant given the high prevalence of myopia worldwide, the diagnostic difficulties when glaucoma and myopia coexist, and the diffusion of surgical procedures to correct refractive errors. The authors confirmed that disc hemorrhage is strongly associated with structural progression of the disease and that IOP fluctuations—and not mean IOP (which is modified by refractive surgery when measured with standard Goldmann tonometry)—are associated with the progression of the disease just in patients who had received refractive surgery. An intense follow-up of these challenging patients is mandatory to prevent severe progression of glaucoma. Normal tension glaucoma is a very subtle, frequently misdiagnosed disease. Jeon et al. [6] showed that the function of the renin–angiotensin–aldosterone system is impaired in these patients, with increased renin mean value and variation. In a multivariate analysis, only increased renin variation was associated with this form of glaucoma.

The remaining seven papers explored different therapeutic options for glaucoma. Interestingly, most of these studies focused on surgical management, a fact that clearly reflects the shift from medical to surgical indications occurring in many glaucoma patients in the attempt of guaranteeing them a better quality of life. 

Treatment-related side effects and quality of life were explored in three papers. Park et al. performed a randomized trial to explore the changes occurring in the macular thickness of patients treated with prostaglandin analogues (PGA) and receiving uncomplicated cataract surgery [7]. The authors found similar macular thicknesses in this group of patients compared with a group who discontinued PGA and a control group. They therefore concluded that discontinuing PGA is not useful in the prevention of macular edema after uncomplicated cataract surgery, provided that anti-inflammatory drops are used for the first month postoperatively. A review by Zgryźniak et al. confirmed the efficacy of selective laser trabeculoplasty (SLT) in open-angle glaucoma and ocular hypertension [8]. The authors concluded that, given the optimal balance between IOP-lowering effect and side effects, SLT should be a first-line treatment, switching to other treatment modalities when target IOP is not achieved. We published a paper prospectively comparing the ocular surface status in patients who had received trabeculectomy on one eye and medically treated in the other eye [9]. We showed that glaucoma surgery is associated with better homeostasis of the ocular surface (less hyperemia and corneal staining, and a stabler tear film). Corneal staining was present in 15 of 26 medically treated eyes and just on 9 eyes after surgery. If we consider that patients with bilateral corneal staining had threefold worse questionnaire scores compared with patients with unilateral or no staining, a successful trabeculectomy may be the appropriate choice for decreasing side effects and for increasing the quality of life of these patients.

In their prospective randomized trial, Kopsinis and coauthors elegantly showed that the intraoperative use of bevacizumab obtains similar efficacy to mitomycin C (MMC) during trabeculectomy [10]. Overall, bevacizumab may have a better safety profile; even though postoperative complication rates were higher in MMC group, the difference was not statistically significant. Unfortunately, the authors did not report data on the ocular surface status in the two groups.

Finally, three papers evaluated the clinical impact of minimally invasive glaucoma surgery (MIGS) devices. The efficacy of XEN, one of the most commonly used MIGS devices, was explored by two papers. Lewczuk and coauthors [11] retrospectively compared the IOP-lowering effects in naïve patients and in those who had previously received glaucoma surgery: in both groups, a significant IOP reduction was achieved, even though the effect reduced over time. I personally appreciated the survival curves of qualified and complete success shown in this paper. The efficacy and safety of this device was also summarized by a meta-analysis by the group of Poelman [12]. The group of Prof. Figus retrospectively explored the efficacy of another device (Ex-PRESS) combined with cataract removal and an everting suture technique in the management of angle-closure glaucoma on 23 eyes [13]. The success of this technique was shown even though the survival curves would suggest that MIGS may not be the ideal choice when a strong, long-term IOP-lowering effect is needed.

Several interesting findings are derived from this collective body of work. It is clear that, today, glaucoma experts are focused on preserving not only the visual function but also the quality of life of patients. This is possible thanks to the better comprehension of detrimental factors affecting the quality of life of people with glaucoma as well as the introduction of less invasive laser and surgical options. Additionally, diagnostics are becoming progressively more effective, but there are still many questions that need more evidence in order to be answered, for example, the role of the integration between morphology and function, or the impact of big data analyses on glaucoma. Considering the limited resources and the dramatic increase in glaucoma patients that we will have to manage in the future, new models of care should be explored, including screening of high-risk subjects and a more widespread use of telemedicine. As the Guest Editor, I give special thanks to the reviewers for their professional comments; in many cases, their volunteer work was valuable to improving the quality of the papers. I am also grateful to the *JCM* team for their robust support. Finally, I sincerely thank all of the authors for their valuable contributions.
